# The New Orleans Food System and COVID-19: A Case Study in Strengthening Food System Resiliency to Facilitate Healthy Eating

**DOI:** 10.3390/nu17233689

**Published:** 2025-11-25

**Authors:** Brandi Stein, Megan Knapp, Elisa Muñoz, Donald Rose

**Affiliations:** 1 School of Health Promotion and Kinesiology, Texas Woman’s University, Denton, TX 76204, USA; bstein1@twu.edu; 2 Department of Public Health Sciences, Xavier University of Louisiana, New Orleans, LA 70125, USA; mknapp@xula.edu; 3 New Orleans Food Policy Action Council, New Orleans, LA 70124, USA; elisa@nolafoodpolicy.org; 4 Department of Social Behavioral and Population Sciences, Celia Scott Weatherhead School of Public Health and Tropical Medicine, Tulane University, New Orleans, LA 70118, USA

**Keywords:** food system, food policy, healthy eating, COVID-19 response, community partnerships

## Abstract

**Background/Objectives:** Policies to promote healthy eating often work through local food systems, which link food supply chains and food environments to individuals. The COVID-19 pandemic exposed vulnerabilities in national and global supply chains and emphasized the importance of local food systems in meeting community and individual needs. Unfortunately, we know too little about how to shape local food systems. This case study reports the impacts of the COVID-19 pandemic on the New Orleans food system and the subsequent response from the community and local government to strengthen it through city and state policy changes, public–private collaborations, and grassroots citizens’ efforts. **Methods:** This study uses a participant–observer approach in which observations from an online 2020 survey of local food organizations (*n* = 56) were fielded by the New Orleans Food Policy Action Council (FPAC), a local coalition of food and agriculture groups. The authors, who worked with or were a part of FPAC, analyzed survey data for recurrent themes and then synthesized this with archived written materials and the authors’ own observations. **Results:** Key themes from this survey are detailed, along with an exploration of related efforts within the community, including: (1) Greaux the Good, a campaign developed by local food system stakeholders to successfully advocate for increased food assistance funds from the Supplemental Nutrition Assistance Program; (2) policy change related to food vendor permitting; and (3) establishment of two positions within the local government: a City Food Specialist to increase collaboration between organizations in the food system and an Urban Agriculture Liaison to support local food production. Lastly, innovative programs of grassroots citizens’ organizations are detailed due to their positive impact on food access for community members. **Conclusions:** The cumulative impacts of these efforts added to the resiliency of the local food system and may protect it against the effects of future disasters as well as strengthen its ability to promote healthy eating.

## 1. Introduction

The COVID-19 pandemic was a worldwide crisis unlike any experienced in the past century. Globally, over 7 million individuals died from the disease, and over 1.2 million died in the United States alone [1]. In addition to the toll on human life, many social systems were disrupted, including transportation, education, employment, and food systems. Problems in food systems were notable from early in the pandemic. Supply chain disruptions kept food from reaching supermarkets, food pantries, and food banks, while the economic impacts of the pandemic decreased donations and increased food insecurity early in the pandemic [2]. Food loss at the farm level increased due to restrictions on the mobility of seasonal farm workers, the decreased movement of produce to markets [3], and a sharp decrease in restaurant sales, which left producers without markets to sell their food [4].

Creating a local food system that is strong and resilient is necessary for overcoming such crises and ensuring the well-being of the community. For a local food system to be sustainable, each sector (food production, manufacturing, distribution, retailing, and consumption) must work in equilibrium to ensure that the local or regional community has continued access to safe and adequate foods while the food system remains profitable and avoids any negative impact on its environment [5].

This proved challenging for local food systems throughout the COVID-19 pandemic [6,7], but even in the absence of crises, local food systems regularly face challenges. Local food producers face barriers that include inadequate storage or processing space, lack of local food retailers to sell local foods, high costs to transport products to market, and insufficient supply chain partnerships to reach institutions and indirect markets [8,9]. Other challenges, such as limited produce donations to food pantries [9], neighborhoods with inadequate access to healthy foods [10,11,12], and insufficient household income, are commonplace [13]. Each of these considerations limits community members’ ability to engage in health-promoting diet-related behaviors.

The strain that the COVID-19 pandemic placed on the food system reinforced the severity of these barriers. While these issues are not new to the food system, the COVID-19 pandemic served as a “wake-up call” that emphasized the vulnerabilities food systems may face in crises. And such crises are likely to come. An analysis of the intensity and frequency of pandemics in recent history shows that the likelihood of experiencing another global pandemic in one’s lifetime is 38% [14]. This increased risk of pandemics is driven by climate change, loss of biodiversity, human-caused pollution, and population growth [15], all of which directly affect food systems, further emphasizing the importance of strengthening them.

How can resilience be improved in a local food system? What do local stakeholders have to say about these concerns? How can improving local food system resiliency contribute to healthy food consumption? These questions are at the core of research gaps in this field. To address these gaps, this descriptive case study identifies the needs of the New Orleans community and the steps taken by local government, food system stakeholders, and local organizations to create meaningful change in the local food system and increase access to foods that contribute to a healthy diet. In documenting these developments, our objective is to draw out lessons learned and enhance the conversation about building resilient food systems. New Orleans, like many cities in the country, has a food policy council and a strong local food system. It was also an early epicenter of the COVID-19 outbreak. All of these characteristics make it a useful place to situate this case study.

### 1.1. The Pre-COVID-19 New Orleans Food System

The New Orleans food system had its own strengths and weaknesses that existed before the COVID-19 pandemic. Louisiana’s food production sector includes fisheries and aquaculture, rice, sugarcane, sweet potatoes, and livestock [16], all of which have fueled the culinary scene of New Orleans. Despite the vibrant food culture, residents of New Orleans face food insecurity rates well above the national average [17,18]. Additionally, New Orleans and the surrounding region are often affected by natural disasters, the most significant in recent history being Hurricane Ida, and before that, Hurricane Katrina. These natural disasters impact all sectors of the food system, from production to consumption. It is estimated that from 2000 to 2011, the economic impact of natural disasters on Louisiana’s agriculture sector reached almost 5 billion [19]. One year after Hurricane Katrina, the number of supermarkets was less than half of pre-Katrina levels [20]. This led to decreased food access for all New Orleans residents for a number of years and disproportionately impacted food access in predominantly Black neighborhoods [21,22]. After Katrina, the food system was strengthened with the addition of food-related organizations that focused on gardening and farming, food distribution, nutrition education, and sustainability [23,24].

### 1.2. The COVID-19 Pandemic in New Orleans

The SARS-CoV-2 virus had a swift impact on the City of New Orleans. The first confirmed case took place on 9 March 2020, and within one month, the number of confirmed cases reached 5242 [25]. At one point, this initial onslaught of confirmed cases in Louisiana had the fastest growth in the world [26]. This led to policy enactments on the state and local level that aimed to slow the spread of the virus and, in doing so, impacted several aspects of the food system.

The first year of COVID-19 in New Orleans saw three distinct waves. In the first wave (March to June 2020), a stay-at-home order was enacted on March 16th [27]. This resulted in the closure of all non-essential businesses, social distancing mandates, and a prohibition on seating customers at restaurants. In mid-June, the city then loosened the policy, allowing restaurants and other non-essential businesses to open with a 50% capacity limit [28]. The city progressively relaxed restrictions throughout the summer and fall of 2020, including increasing the capacity and limits of restaurants. A spike in cases from November 2020 to January 2021, the third wave, led to a sharp increase in restrictions that mandated no gatherings and special events and reduced indoor activities to 25% capacity [29]. Fluctuations in restrictions continued to occur following trends in positive COVID-19 tests and hospital admission rates throughout the city.

### 1.3. Impacts of COVID-19 on the New Orleans Food System

The COVID-19 pandemic had a negative economic impact on many businesses and people in New Orleans. The New Orleans Business Alliance found that of the nearly 60,000 accommodations, food service, and retail sales jobs in New Orleans, over 50% of them were lost during the COVID-19 pandemic [30]. Additionally, while grocery store and pharmacy visits decreased by 32% at the beginning of the pandemic, retail and restaurant visits were 68% lower than their pre-pandemic levels at one point. A year and a half after the pandemic began, the number of visits to either of these sectors had not recovered to pre-COVID-19 levels.

Each sector of the food system was impacted by COVID-19. In addition to the retail and food service sectors as described above, food production, consumer goods, food pantries and feeding sites, food system technical assistance providers, food waste and recovery, and distribution were affected. The New Orleans Food Policy Action Council (FPAC), a broad-based coalition of individuals, organizations, communities and businesses working together to support equitable food and agriculture policy, sought to understand the needs of each sector and advocated for policy change and funding to support the needs of both local food system stakeholders and community members. FPAC also wanted to gain a better understanding of what actions food-related organizations and businesses were taking to mitigate the effects of the pandemic and meet the nutrition-related needs of the community.

## 2. Materials and Methods

In 2020, to address these information needs, FPAC surveyed businesses and organizations representing various sectors of the New Orleans food system, including production, distribution, consumption and access, and food waste and recovery. Modeled after the Community Food System Assessment Framework [31] used to study the local food environment and based on the experiences of the authors, the survey asked 16 general questions to all respondents and utilized a skip pattern for an additional 7 to 20 questions tailored to the food sector represented by the respondent. The questions focused on the impact of COVID-19 on operations, resources utilized, organizational needs, partnerships, and innovations. Of the 16 general questions, 9 were open-ended, providing information in the respondents’ own words. See Appendix A for the full instrument. The survey was administered using the online software Research Electronic Data Capture (REDCap) version 9.8.2. Participants were given $15 for survey completion.

The initial survey invitation was distributed via email to 73 members of FPAC and was shared via the coalition’s social media accounts. In total, 60 individual organizations participated in the survey. Three responses were removed due to incomplete information, and one response was removed due to its later categorization outside of the food sector (i.e., skin care), resulting in 56 responses that were analyzed for a response rate of 76.7%. These organizations come from all sectors of the food economy, from production to consumption to waste management.

Quantitative responses were summarized using descriptive statistics. Open-ended responses were extracted verbatim into a spreadsheet and read by all authors. An inductive approach was used to identify segments of responses with codes, such as community partnerships or public support. The lead author collated these codes and examined them for emerging themes, such as the importance of community partnerships for organizational survival or the utility or limitations of public support. All authors met repeatedly to review these key themes, refine their clarity, and prepare the written summaries.

We describe our methods used in this work as a participant–observer approach [32], in part because of the positioning of the authors and because of the way we integrated various strands of evidence. One of our authors (EM) was the executive director of FPAC, which provided the sample of food organizations surveyed here and which had an active role in policy advocacy at the city level. The lead author (BS) conducted the practicum experience for her master’s degree with FPAC. Another author (MK) is on the steering committee of FPAC, and the last author (DR) was involved in the initial founding of FPAC. Both MK and DR continue to work with FPAC and send their students there for experiential learning.

The research team synthesized recurrent themes from this survey data with archived written materials and the authors’ own observations to understand the process of advocacy for local policy change and support for public–private partnerships. All authors participated in the food system, both professionally and personally, being citizens of New Orleans. Our observations in these capacities helped us to develop a relevant questionnaire and allowed us to better interpret themes that emerged. We combined this with our own notes on policy advocacy efforts that were made. We also cross-checked our information with public sources on new programs, such as the State of Louisiana and City of New Orleans websites. An exploration of additional grassroots interventions was conducted using our own knowledge of these activities, complemented by source material from organizational websites (e.g., for Krewe of Red Beans). This resulted in a comprehensive case study analysis of the efforts made to strengthen the resiliency of the local food system and community members’ access to healthy foods in response to the COVID-19 pandemic.

## 3. Results

### 3.1. Survey Respondent Observations

Survey participants represented multiple sectors of the food system, including food production, food service, consumer goods, food pantry or feeding site, food system technical assistance, food waste and recovery, and retail and distribution (see Table 1). Of the 56 organizations surveyed, 51.8% remained in business during the pandemic, 17.9% temporarily closed and then reopened during the pandemic, and 8.9% remained closed. The remaining 21.4% of businesses did not specify how the pandemic impacted operational status.

Of the 17 organizations that were identified primarily as food producers, 43% reported losing a consumer-facing market such as retail, restaurants, or catering. However, 63% of food producers also reported gaining a new market, often in the form of an outdoor farmers' market. Of the 34 small businesses surveyed, 24% reported having to let go of staff due to the pandemic, and 53% reported an increase in the cost of operations for their business. At the time, there was continuous uncertainty regarding the trajectory of the pandemic, and respondents reported ongoing concerns such as funding for small businesses, confusion on permitting requirements, and safe food access for community members.

#### 3.1.1. Community Partnerships

Many of these respondents credited community partnerships for their ability to launch new ventures and access new markets. One food pantry operator relayed, “We were able to mobilize quickly because of our existing relationships and find more creative solutions […] because of those partners”. A local food producer saw benefit in their partnerships as well, stating, “Finding new revenue sources through community partnerships such as drive through farmers markets has been helpful”. These community partnerships extended beyond the food sector. Another food pantry operator found, “Community partnerships have helped us get connected to additional resources to share with our clients, including hurricane kits from a partnership”. These collaborations led to a larger network of organizations that worked together to meet the needs of the community. It allowed organizations to see what was working for others and replicate it, while also minimizing overlap in services provided to the community. Community residents were attracted to these new services, such as the drive-through farmers’ market, which offered them a new opportunity to access healthy food during the pandemic.

#### 3.1.2. Strengths and Limitations of Public Support

Respondents emphasized the importance of support from both the federal and local governments for navigating the pandemic. One respondent from a local food bank noted how logistical and financial support allowed them to meet the food and nutrition needs of the community, stating, “New government programs […] have provided additional food to distribute. We’ve transitioned some of our work to a ‘mass distribution’ model, allowing us to serve thousands of families in no-contact food distribution events. The assistance of the National Guard and volunteers has been crucial for running these events successfully. Financial resources have allowed us to expand equipment and staff, making it possible to increase our services by 56% in response to the pandemic”. Some respondents reported that navigating government programs and funding was complicated and sometimes lacked transparency. A local food retailer noted how a local community-based organization stepped in to help connect organization leaders to reach the appropriate resources, reporting, “[Local community-based organization], in particular, was very helpful to its alumni in helping us navigate the government aid application process”.

Other respondents shared the need for access to these kinds of opportunities. One food pantry operator emphasized the problems that small food pantries were facing, stating, “Getting assistance to run these pantries and actually investing money into this work—which has a huge impact—would be a big deal. We also need major players […] to offer more opportunities for pantry operators to connect, train on other resources like SNAP, and share solutions with one another”. An urban agriculture organization leader noted the importance of investing in local food-related businesses, sharing, “I need the city government to recognize that food production enterprises create a more resilient city and contribute to public health and economic revitalization and accordingly to invest in food production enterprises”. These comments emphasized the need for investment in local food businesses for both short- and long-term ventures to build a robust local food system, as these have direct impacts on community health. They also highlight the opportunity for public–private partnerships in connecting organizations, government officials, and other entities that are focused on food and nutrition access in the community.

#### 3.1.3. Importance of Grassroots Organizing

Some respondents were less optimistic about assistance from the local government. Another urban agriculture organization leader noted, “City government is not 100 percent invested in local food distribution by local farmers […]. All of the local food distribution is successful because of the not-for-profit organizations, grass roots [organizations] and farmers working together”. Some felt that the government policies were not adequate in meeting New Orleans’ unique needs. Another referenced the extensive documentation requirements needed for individuals to access food aid provided from federal relief funds. This perceived lack of support from official avenues may have prompted grassroots organizations to establish programs to support mutual aid efforts throughout the city.

FPAC, leaders, and other community organizations heard these needs and acted through policy changes, public–private collaborations, and grassroots efforts.

## 4. Discussion

### 4.1. Policy Changes

In Louisiana, the number of individuals eligible for the Supplemental Nutrition Assistance Program (SNAP) increased by approximately 30% in 2020 [33]. Additionally, respondents who serve as local food producers reported a need for financial resources and government assistance. In response to these needs, local stakeholders developed the Greaux the Good campaign to advocate for a bill that provides funding for local farmers' markets to create or expand a market match program [34] that would allow SNAP food assistance participants to receive double the amount of benefits when shopping at Louisiana farmers’ markets. The campaign argued that funding a market match program would strengthen the local food system by increasing funding and promotion for local farmers to grow and sell food, as well as increasing healthy food access and consumption within the community. Bill HCR57 passed in 2022, and the Greaux the Good market match program is operated through Market Umbrella, a local organization that administers various farmers' markets and provides technical assistance to markets throughout Louisiana. Although this program was initiated as a response to the COVID-19 pandemic, it has been funded for a third year using funds from Louisiana’s state budget, totaling nearly $3 million in investment.

Throughout this period, the city also took actions to support the culinary sector of New Orleans in innovative ways. This included starting the Outdoor Dining Program that allowed businesses to expand their outdoor dining options by using the sidewalk or street in front of their business. It also provided businesses with funding to create this expanded outdoor dining space [35]. Local food businesses adapted to the COVID-19 pandemic by starting pop-up restaurants at local outdoor venues throughout the city. However, the permitting process was both logistically and financially burdensome. To host a pop-up restaurant event, a vendor was required to obtain seven individual permits totaling upwards of $1400. In response to advocacy by FPAC and other food system stakeholders, the city government simplified the process, requiring that the pop-up vendor and pop-up host each acquire one $100 permit. Local government also created the City of New Orleans One Stop Permits and Licenses application to streamline the permitting process [36]. However, lack of staffing to review and approve licenses has served as a barrier to local businesses acquiring permits. This has led to pop-up restaurant vendors receiving citations despite their best efforts to acquire the appropriate permits needed to function. The city is also developing an Emerging Vendor Permit for new food businesses that are not associated with already established venues and are interested in innovative vending practices. The Emerging Vendor Permit is set to be approved in 2025.

### 4.2. Public–Private Collaboration

During the COVID-19 pandemic, respondents identified community partnerships between food-related organizations as factors that strengthened the resiliency of the food system, a sentiment that was echoed in other regional food systems [7,37]. In New Orleans, this encouraged local food system stakeholders to advocate for a City Food Specialist position with the goal of addressing food issues at the city level and strengthening community partnerships and coordination. Funding was acquired for the position, and the position was established and has been filled within the New Orleans Health Department. Since 2023, this position has been funded by FPAC but operated within the New Orleans Health Department; in October 2025, the city took on funding of the position after realizing the partnerships and capacity it brought to the department. This position continues to serve on the FPAC steering committee and co-chairs the FPAC Disaster and Food Access working groups. Patience and persistence were key aspects to making this happen. Although efforts to create this food-specific position within the city started as early as 2009, it was not filled until 2024.

In addition, New Orleans growers had unique needs to address. Through A Greener NOLA policy campaign, FPAC and urban agriculture partners Sprout and Greater New Orleans Growers Alliance identified a need for an Urban Agriculture Liaison to sit within the city government. Through relationship building, community organizing, and advocacy to policymakers, this position was added to the City of New Orleans’ 2024 operating budget and now sits in the Office of Resilience and Sustainability. A Greener NOLA partners collaborated on aspects of this position, including the job description, hiring rubrics, and community accountability, again showing the need for strong collaboration and coordination with public and private entities. In 2025, through this position, multiple policy changes were enacted to encourage urban agriculture in New Orleans.

While many of the actions taken were based on longstanding recommendations, the COVID-19 pandemic provided an urgency to act as well as opportunities for funding through the American Rescue Plan Act (ARPA) and increased philanthropic support to help with recovery.

### 4.3. Grassroots Efforts

Local organizations reacted swiftly and creatively to support community members as the COVID-19 pandemic emerged. Crescent City Farmers Market (CCFM) was able to pivot quickly at the onset of the pandemic, setting an example for how farmers' markets can still support vendors and consumers. CCFM switched to a drive-thru market model to allow consumers to safely receive groceries. Additionally, they paired with Top Box Foods, a local food distribution organization, to provide food directly from the farmers' market vendors to the homes of New Orleanians.

The Krewe of Red Beans, a local Mardi Gras club and social support organization, created jobs and fed local first responders and seniors through their “Feed the Front Line” and “Feed the Second Line” grocery delivery programs [38,39]. Initially, Feed the Front Line was developed to provide meals to health care workers on the “front line” of the pandemic. The meals were purchased from local restaurants and delivered by local musicians. This dynamic provided restaurants with the business they needed to stay afloat and musicians with an income while they were out of work due to the restrictions on live music. This program was followed by Feed the Second Line, which aimed to protect a group of elder “culture bearers” from the COVID-19 virus by again hiring local musicians to purchase and deliver groceries to a high-risk group of individuals [38]. This program has expanded into much more. They also partnered with local businesses and organizations to create the Get Lit, Stay Lit program. Inspired by extensive and lengthy electrical outages from Hurricane Ida, this program aims to train local individuals in the skills needed to install solar panels on participating restaurants in the New Orleans area. When future disasters occur, these restaurants will be able to serve as hubs for food and water, charging stations, and cooling centers [39]. Feed the Second Line continues to purchase groceries for community members at the time of publication.

These efforts highlight how dedicated New Orleans organizations are to meeting the food and nutrition needs of the community during and beyond crises. Through innovative programming, these organizations continue to meet the needs of the community and build capacity to mitigate the impacts on food access during food system disruptions.

### 4.4. Limitations

There are a number of limitations to our research, which may affect its generalizability. First, our research used a participant-observer approach. To draw our sample, we started with an organizational list based on members of FPAC, which might have resulted in a sample selection bias. An outside research entity may have had a more objective approach, chosen a different sample, and come to differing conclusions. However, it is often difficult to gain the trust of non-profit organizations, and this was particularly true during the pandemic. This common challenge was easier for us to overcome, given our connections with these organizations. Moreover, New Orleans can be an enigmatic city. Our understanding of the place, based on years of residence, allowed us to decipher some of the nuances. This underscores another limitation, namely that our research is based on just New Orleans, which has a very distinct geography and cultural landscape, especially its well-known food culture.

However, despite its uniqueness, there are many aspects of New Orleans that are common to other cities, and for which insights from this paper can be gleaned. All cities have local food systems that include elements of the food chain, from food production to consumption, and many of them were affected by the pandemic, as was New Orleans. Furthermore, many cities have local food policy councils that interface between the food sector and local and state government. The development of policies based on the needs expressed by local organizations and the linkage of the food sector to local governments through new employee positions were two developments in New Orleans that could translate to other areas.

## 5. Conclusions

The COVID-19 pandemic exposed the gaps in the food system and acted as a catalyst for discussions on policy change, funding, and other opportunities to support the local food system. Individuals and organizations within the food system acted innovatively to adapt to challenges presented by the COVID-19 pandemic. By identifying and understanding the needs of the community, stakeholders in the food system proposed and supported programs and policy changes to increase the food system’s resiliency. Local food-related organizations pivoted their business strategy and operations, increased efforts to support the community, and collaborated with other organizations to support each other and the community. New Orleans FPAC established two new positions within city government, local stakeholders successfully advocated for policy change related to SNAP and local permitting, and grassroots organizations collaborated to support the changing needs of the community. While improvements to the local food system were made, many stakeholders and local business owners agree that further efforts are required to reach a level of resiliency that protects the people and organizations that make up the New Orleans food system. Additional research on local food system resiliency to monitor these interventions over the long term and to compare them with developments in other cities is also needed. This is especially the case in light of changing political and economic contexts.

## Figures and Tables

**Table 1:** Types of Organizations Surveyed (*n* = 56).

Organization Type ^1^	Examples	*n* (%)
Food Production	Farms, urban gardens	17 (30.4)
Food Service	Restaurants, food trucks	14 (25.0)
Consumer Goods	Canned foods, bakery items	8 (14.3)
Food Pantry or Feeding Site	Food banks, community fridges, school to-go meals	7 (12.5)
Food System TA	Gardening assistance, nutrition education	6 (10.7)
Food Waste and Recovery	Composting, food redistribution	2 (3.5)
Retail Distribution	Grocery markets, delivery services	2 (3.6)

^1^ Organizations were categorized based on qualitative survey responses that identified the primary interventions and efforts of the organization.

## Data Availability

The raw data supporting the conclusions of this article will be made available by the authors on request to protect participant privacy.

## Appendix A. Food System COVID-19 Response Survey

1. *Type of organization (Select all that apply)*
  a. *Small Business*
  b. *Food Pantry*
  c. *Emergency Feeding Site*
  d. *School Feeding Site*
  e. *Food Waste and Recovery*
  f. *Food Production*
  g. *Other*
2. *How has COVID-19 impacted your business?*
  a. *My business has remained open*
  b. *My business has closed*
  c. *My business has opened during COVID-19*
  d. *Other*
3. *What is the zipcode served by your site/business/organization?*
  *__________________________________*
4. *What resources have helped you the most during COVID-19? (Select all that apply)*
  a. *Financial Resources*
  b. *Community Partnerships*
  c. *Government Assistance*
  d. *Mutual Aid Partners*
  e. *Volunteers*
  f. *Other*
5. *How have these partnerships helped? __________________________________________*
6. *What new connections have been made that have allowed you to continue work during COVID-19? (Please respond N/A if this does not apply to you)*
  *__________________________________________*
7. *How have your previous connections helped your site/business/organization succeed during COVID-19? (Please respond N/A if this does not apply to you)*
  *__________________________________________*
8. *If your business is still in operation, do you anticipate that you will be able to continue operations if the COVID-19 crisis continues?*
  a. *Yes*
  b. *No*
  c. *Not sure*
9. *What will your site/business/organization need to stay in business? (Please respond N/A if this does not apply to you)*
  *__________________________________________*
10. *What do you need from the City government right now? (Please respond N/A if this does not apply to you) __________________________________________*
11. *Has your organization made or planned any efforts (individual or in collaboration) to combat the COVID-19 crisis?*
12. *What efforts (individual or in collaboration) are being done or being planned in response to the COVID-19 crisis? (Please respond N/A if this does not apply to you)*
  *__________________________________________*
13. *Do you have a separate job from this site/business/organization that has been affected by COVID-19?*
  a. *Yes*
  b. *No*
14. *How has this job been affected by COVID-19?*
  a. *Laid off*
  b. *Work hours reduced*
  c. *Work hours increased*
  d. *No change*
  e. *N/A*
15. *In the longer-term, what are some solutions you think could help our economy operate safely during a global pandemic? __________________________________________*
16. *From your perspective, what role have federal aid programs (for example, SNAP, WIC, P-EBT, TEFAP, enhanced Unemployment Insurance,* etc.*) played for your clients during the pandemic? (Please respond N/A if this does not apply to you)*
  *__________________________________________*

*Food Production*

*Have you lost any markets as a result of COVID-19?*
  a. *Yes*
  b. *No*
  c. *Not sure*
  *Which markets have you lost as a result of COVID-19?*
  *__________________________________________*
*Did you gain any new markets after 15 March 2020?*
  a. *Yes*
  b. *No*
  c. *Not sure*
  *What new markets did you gain after 15 March 2020?*
  *__________________________________________*
*Did your sales increase after 15 March 2020?*
  a. *Yes*
  b. *No*
  c. *Not sure*
  *What were your estimated weekly sales BEFORE 15 March 2020?*
  *__________________________________*
  *What were your estimated weekly sales between 15 March 2020, and today?*
  *__________________________________*
  *How many estimated pounds did you sell weekly BEFORE 15 March 2020?*
  *__________________________________*
  *How many estimated pounds did you sell weekly AFTER 15 March 2020?*
  *__________________________________*

*Small Business*

*Type of small business(Check all that apply)*
  a. *Restaurant*
  b. *Catering*
  c. *Food Truck*
  d. *Consumer Packaged Goods/Value Added Product*
  e. *Retail (ie. grocery store/corner store)*
  f. *Incubator/Commercial Kitchen*
  g. *Distributer*
  h. *Farm*
  i. *Other*
*Have you lost a specific client base or market during COVID-19?*
  a. *Yes*
  b. *No*
  c. *Not sure*
  *What client base or market have you lost since 15 March 2020?*
  *__________________________________________*
  *Approximately what percentage (%) of business have you lost since 15 March 2020?*
  *__________________________________*
  *How many active staff members did you have BEFORE 15 March 2020?*
  *__________________________________*
*Has your business had to let go of staff since 15 March 2020, due to COVID-19?*
  a. *Yes*
  b. *No*
  c. *Not sure*
  *How many staff members have been let go since 15 March 2020, due to the COVID-19 pandemic?*
  *__________________________________*
*Have the costs of operating your business increased since 15 March 2020, due to COVID-19?*
  a. *Yes*
  b. *No*
  c. *Not sure*
*What costs have increased since 15 March 2020?*
  a. *Operation Cost/Staff*
  b. *Shipping/Delivery*
  c. *Food/Supplies*
  d. *Other*
*Have you experienced a supply chain disruption that has halted business and profit since 15 March 2020?*
  a. *Yes*
  b. *No*
  c. *Not sure*
  *What supply chains have disrupted the operations of your business since 15 March 2020?*
  *__________________________________________*
*Were you able to access any loans or financial services during COVID-19?*
  a. *Yes*
  b. *No*
  c. *Not sure*
*Have you applied for the Payroll Protection Program (PPP) loan?*
  a. *Yes*
  b. *No*
  c. *Not sure*
*Have you received any portion of the Payroll Protection Program (PPP) loan?*
  a. *Yes*
  b. *No*
  c. *Not sure*
*Have you applied for the Economic Injury Disaster Loan (EIDL)?*
  a. *Yes*
  b. *No*
  c. *Not Sure*
*Have you received any portion of the Economic Injury Disaster Loan (EIDL)?*
  a. *Yes*
  b. *No*
  c. *Not sure*
*Were there any organizations or community partners that helped with financial services, supply chain, food procurement, and PPP disruption due to COVID-19?*
  a. *Yes*
  b. *No*
  c. *Not sure*
  *If you answered “Yes” to the question above, what partnerships did you gain during COVID-19 that impacted your business? __________________________________________*
*Has COVID-19 made you more or less interested in entrepreneurship?*
  a. *More*
  b. *Less*
  c. *No change*
*Have you started working on a new business or business concept, motivated by/as a result of COVID-19?*
  a. *Yes*
  b. *No*
  *Food Waste and Recovery*
*What is your relationship with the food waste and recovery business?*
  a. *Distributor*
  b. *Pickup*
  c. *Process and distribute*
  d. *I have my compost picked up or distributed by a partner*
  e. *Does your operation collect food waste?*
  f. *Yes*
  g. *No*
  h. *Not sure*
*What type of food waste is collected? (Select all that apply)*
  a. *Surplus edible foods*
  b. *Food for animals*
  c. *Food for compost*
  *How many pickup locations did you operate BEFORE 15 March 2020?*
  *__________________________________*
  *How many pickup locations did you operate AFTER 15 March 2020?*
  *__________________________________*
  *How many partners (restaurants, businesses, schools, individuals) have you lost pickup to since 15 March 2020?__________________________________*
  *How many partners (ex: restaurants, businesses, schools) have you gained pickup to since 15 March 2020? __________________________________*
  *How many estimated pounds were collected weekly BEFORE 15 March 2020?*
  *__________________________________*
  *How many estimated pounds were collected weekly AFTER 15 March 2020?*
  *__________________________________*

*Food Access—School Meals*

*When did your school meals site open for COVID-19 relief?*

*__________________________________*

*When did your school meals site end for the year? __________________________________*

*What were your operating hours BEFORE 15 March 2020?*

*__________________________________*

*What were your operating hours AFTER 15 March 2020?*

*__________________________________*

*How many estimated meals were served daily at your site BEFORE 15 March 2020?*

*__________________________________*

*How many estimated meals are being served daily at your site since 15 March 2020?*

*__________________________________*

*How many estimated snacks were served daily at your site BEFORE 15 March 2020?*

*__________________________________*

*How many estimated snacks are being served daily at your site since 15 March 2020?*

*__________________________________*

*What agencies have you connected with to better meet the needs of the communities being served?*

*__________________________________________*

*What agencies were involved in the sourcing and the operation of your school meal site?*

*__________________________________________*

*Food Access—Food Pantry*

*When did your food pantry program open? __________________________________*

*How many emergency feeding sites does your program operate?*

*__________________________________*

*What services does your emergency feeding program offer?*
  a. *Drive-thru*
  b. *Walk-up*
  c. *Delivery*

*How many estimated families were served at your site this year?*

*__________________________________*

*How many estimated families have been served at your site since 15 March 2020?*

*__________________________________*

*How much money has been spent on food since 15 March 2020?*

*__________________________________*

*How many pounds of food has been served daily since 15 March 2020?*

*__________________________________*

*Food Access—Emergency Feeding*

*When did your emergency feeding site open? __________________________________*

*How many estimated families were served daily at your site BEFORE 15 March 2020?*

*__________________________________*

*How many estimated families are being served daily at your site since 15 March 2020?*

*__________________________________*

*What agencies have you connected with to better meet the needs of the communities being served?*

*__________________________________*

*What were your operation hours BEFORE 15 March 2020?*

*__________________________________*

*What were your operation hours AFTER 15 March 2020?*

*__________________________________*

*How much money was needed to fund this site? __________________________________*

*What agencies were involved in the sourcing and the operation of your emergency feeding site?*

*__________________________________*

## References

[1] World Health Organization (WHO) (2024). Coronavirus (COVID-19) Dashboard. https://data.who.int/dashboards/covid19/deaths?n=c.

[2] Leone L.A., Fleischhacker S., Anderson-Steeves B., Harper K., Winkler M., Racine E., Baquero B., Gittelsohn J. (2020). Healthy Food Retail during the COVID-19 Pandemic: Challenges and Future Directions. Int. J. Environ. Res. Public Health.

[3] Barman A., Das R., De P.K. (2021). Impact of COVID-19 in food supply chain: Disruptions and recovery strategy. Curr. Res. Behav. Sci..

[4] Johnson R., Congressional Research Service (2020). COVID-19: Supply Chain Disruptions in the U.S. Fruit and Vegetable Industry: In Brief. https://crsreports.congress.gov/product/pdf/R/R46348.

[5] Nguyen H. (2018). Sustainable Food Systems: Concept and Framework. https://www.fao.org/3/ca2079en/CA2079EN.pdf.

[6] Galanakis C.M. (2020). The food systems in the era of the coronavirus (COVID-19) pandemic crisis. Foods.

[7] O’Connell C., Gay R., McDonald N., Sita T. (2021). COVID connections: Lessons from adaptations to COVID-19 as strategies for building food system resilience. Cult. Agric. Food Environ..

[8] Godette S., Beratan K., Nowell B. (2015). Print e-mail barriers and facilitators to local food market development: A contingency perspective. J. Agric. Food Syst. Community Dev..

[9] Taylor D.E., Ard K.J. (2015). Food availability and the food desert frame in Detroit: An overview of the city’s food System. Environ. Pract..

[10] Breyer B., Voss-Andreae A. (2013). Food mirages: Geographic and economic barriers to healthful food access in Portland, Oregon. Health Place.

[11] Rose D. (2010). Access to healthy food: A key focus for research on domestic food insecurity. J. Nutr..

[12] Walker R.E., Keane C.R., Burke J.G. (2010). Disparities and access to healthy food in the United States: A review of food deserts literature. Health Place.

[13] Bruce C., Gearing M.E., DeMatteis J., Levin K., Mulcahy T., Newsome J., Wivagg J. (2022). Financial vulnerability and the impact of COVID-19 on American households. PLoS ONE.

[14] Marani M., Katul G.G., Pan W.K., Parolari A.J. (2021). Intensity and frequency of extreme novel epidemics. Proc. Natl. Acad. Sci. USA.

[15] Thoradeniya T., Jayasinghe S. (2021). COVID-19 and future pandemics: A global systems approach and relevance to SDGs. Glob. Health.

[16] United States Department of Agriculture, National Agricultural Statistics Service (2017). 2017 Census of Agriculture—State Data. https://www.nass.usda.gov/Publications/AgCensus/2017/Online_Resources/County_Profiles/Louisiana/cp99022.pdf.

[17] Coleman-Jensen A., Rabbitt M.P., Gregory C.A., Singh A., United States Department of Agriculture, Economic Research Service (2019). Household Food Security in the United States in 2018. https://ers.usda.gov/sites/default/files/_laserfiche/publications/94849/ERR-270.pdf?v=48326.

[18] Feeding America (2020). Map the Meal Gap 2020: A Report on County and Congressional District Food Insecurity and County Food Cost in the United States in 2018. https://www.feedingamerica.org/sites/default/files/2020-06/Map%20the%20Meal%20Gap%202020%20Combined%20Modules.pdf.

[19] Guidry K.M., Pruitt J.R. (2012). Damages to Louisiana agriculture from natural disasters. Choices. https://www.choicesmagazine.org/UserFiles/file/cmsarticle_249.pdf.

[20] Ulmer V.M., Rathert A.R., Rose D. (2012). Understanding policy enactment: The New Orleans Fresh Food Retailer Initiative. Am. J. Prev. Med..

[21] Mundorf A.R., Willits-Smith A., Rose D. (2015). 10 Years Later: Changes in Food Access Disparities in New Orleans since Hurricane Katrina. J. Urban Health.

[22] Rose D., Bodor J.N., Rice J.C., Swalm C.M., Hutchinson P.L. (2011). The effects of Hurricane Katrina on food access disparities in New Orleans. Am. J. Public Health.

[23] Futch J., Kovacs B., Prasertong A., O’Malley K., Rose D. (2020). Fostering equitable food access and healthy eating through social innovation: A description of the New Orleans experience. Curr. Dev. Nutr..

[24] Tulane Nutrition (2018). A Resource Guide to Innovative Food and Nutrition Work in New Orleans.

[25] Weinstein R. (2021). Monitoring the First Year of the COVID-19 Pandemic in Louisiana. https://www.datacenterresearch.org/covid-19-data-and-information/monitoring-the-covid-19-pandemic-in-louisiana/.

[26] Wagner G.A. (2020). COVID-19 Louisiana Response. https://gov.louisiana.gov/assets/docs/covid/govCV19Brief-2.pdf.

[27] City of New Orleans Department of Health (2020). Guidelines for COVID-19 Response and Stay-at-Home Directives. https://nola.gov/NOLAReady/media/Documents/Coronavirus/Guidelines/NOHD-Phase-1-Guidelines-Clarification-5-29-20-Final.pdf.

[28] City of New Orleans Department of Health Guidelines for COVID-19 Reopening Phase Two. 12 June 2020. https://nola.gov/NOLAReady/media/Documents/Coronavirus/Guidelines/NOHD-Phase-2-Guidelines-updated-6-12-20-1400.pdf.

[29] City of New Orleans (2021). City Announces Modified Phase One as COVID-19 Cases and Hospitalizations Reach a Dangerous Rate in Orleans Parish. https://nola.gov/mayor/news/january-2021/city-announces-modified-phase-one-as-covid-19-cases-and-hospitalizations-reach-a-dangerous-rate-in-o/.

[30] New Orleans Business Alliance (2022). Tracking the Economic Impacts of COVID-19.

[31] Martin K., Morales T., American Planning Association (2015). Community Food System Assessments; PAS Memo. https://www.planning.org/publications/document/9121415/.

[32] Jorgensen D.L. (1989). Participant Observation: A Methodology for Human Studies.

[33] Public Affairs Research Council Louisiana Louisiana’s Food Stamp Usage. https://parlouisiana.org/wp-content/uploads/2024/07/Louisianas-Food-Stamp-Usage-1.pdf.

[34] Louisiana House of Representatives (2022). HLS 22RS-1317.

[35] City of New Orleans (2020). City of New Orleans Launches Outdoor Dining Program. https://www.nola.gov/mayor/news/august-2020/city-of-new-orleans-launches-outdoor-dining-program/.

[36] City of New Orleans One Stop App (2023). Get Started. https://onestopapp.nola.gov/.

[37] Dahal D., Schulsler T.M. (2025). Benefits and challenges of collaborative networks addressing food system disruptions during the COVID-19 pandemic. Front. Sustain. Food Syst..

[38] Krewe of Red Beans About Us. https://www.feedthesecondline.org/about.

[39] Krewe of Red Beans Get Lit Stay Lit. https://www.feedthesecondline.org/get-lit-stay-lit.

